# Electroacupuncture attenuates ferroptosis by promoting Nrf2 nuclear translocation and activating Nrf2/SLC7A11/GPX4 pathway in ischemic stroke

**DOI:** 10.1186/s13020-024-01047-0

**Published:** 2025-01-04

**Authors:** Xi-chen Yang, Ya-ju Jin, Rong Ning, Qiu-yue Mao, Peng-yue Zhang, Li Zhou, Cheng-cai Zhang, Yi-chen Peng, Na Chen

**Affiliations:** Yunnan Key Laboratory of Integrated Traditional Chinese and Western Medicine for Chronic Disease in Prevention and Treatment, Key Laboratory of Acupuncture and Massage for Treatment of Encephalopathy, College of Acupuncture, Tuina and Rehabilitation, Yunnan University of Traditional Chinese Medicine, Kunming, China

**Keywords:** Electroacupuncture, Ischemic stroke, Ferroptosis, Nrf2, Nuclear translocation, Oxidative stress

## Abstract

**Objective:**

Electroacupuncture has been shown to play a neuroprotective role following ischemic stroke, but the underlying mechanism remains poorly understood. Ferroptosis has been shown to play a key role in the injury process. In the present study, we wanted to explore whether electroacupuncture could inhibit ferroptosis by promoting nuclear factor erythroid-2-related factor 2 (Nrf2) nuclear translocation.

**Methods:**

The ischemic stroke model was established by middle cerebral artery occlusion/reperfusion (MCAO/R) in adult rats. These rats have been randomly divided into the EA + MCAO/R group, the MCAO/R group, the EA + MCAO/R + Brusatol group (the inhibitor of Nrf2), and the EA + MCAO/R + DMSO group, and the Sham group. The EA + MCAO/R group, EA + MCAO/R + Brusatol group, and the EA + MCAO/R + DMSO group received EA intervention 24 h after modeling for 7 consecutive days. The behavioral function was evaluated by Neurologic severity score (NSS), Garcia score, Foot-fault Test, and Rotarod Test. The infarct volume was detected by TTC staining, and the neuronal damage was observed by Nissl staining. The levels of Fe^2+^, reactive oxygen species (ROS), superoxide dismutase (SOD), and malondialdehyde (MDA) were measured by ELISA. The immunofluorescence and Western blotting were used to detect the expression of Total Nrf2, p-Nrf2, Nuclear Nrf2, and Cytoplasmic Nrf2, and the essential ferroptosis proteins, including glutathione peroxidase 4 (GPX4), solute carrier family 7 member 11 (SLC7A11) and ferritin heavy chain 1 (FTH1). The mitochondria were observed by transmission electron microscopy (TEM).

**Results:**

Electroacupuncture improved neurological deficits in rats model of MCAO/R, decreased the brain infarct volume, alleviated neuronal damage, inhibited the Fe^2+^, ROS, and MDA accumulation, increased SOD levels, increased the expression of GPX4, SLC7A11 and FTH1, and rescued injured mitochondria. Especially, we found that the electroacupuncture up-regulated the expression of Nrf2, and promoted phosphorylation of Nrf2 and nuclear translocation, However, Nrf2 inhibitor Brusatol reversed the neuroprotective effect of electroacupuncture.

**Conclusion:**

Electroacupuncture can alleviate cerebral I/R injury-induced ferroptosis by promoting Nrf2 nuclear translocation. It is expected that these data will provide novel insights into the mechanisms of electroacupuncture protecting against cerebral I/R injury and potential targets underlying ferroptosis in the stroke.

**Graphical Abstract:**

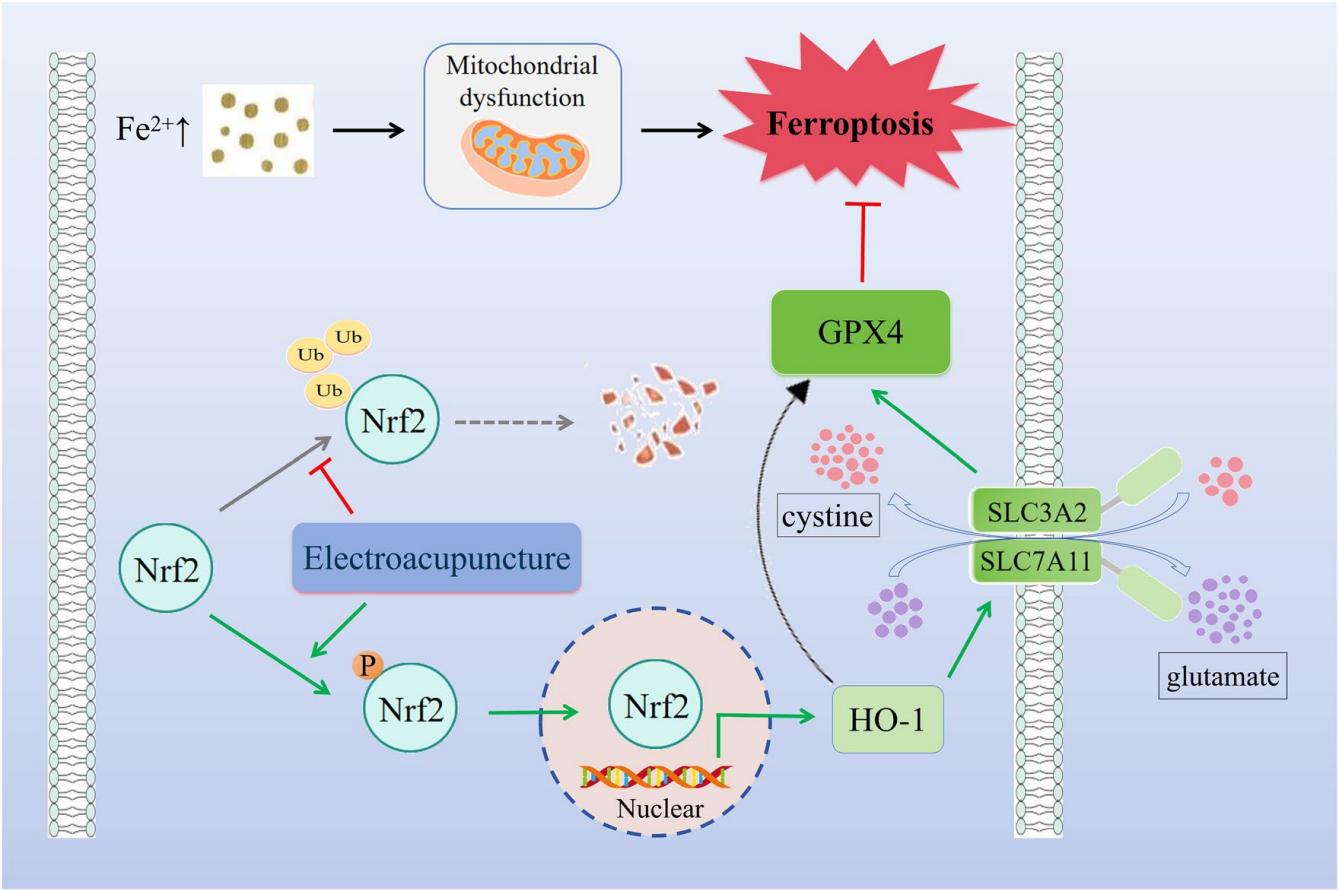

## Introduction

A stroke is caused by an arterial blockage or hemorrhage in one part of the brain tissue, which leads to focal neurological damage in the adjacent area. Ischemic stroke is induced by ischemia and hypoxia in the brain, which leads to limited ischemic necrosis or softening of the brain tissue, and it will cause severe neurological deficits, which can result in cell death if reperfusion is not possible in a short period, this type of stroke accounts for about 70% of all strokes [1]. The incidence and prevalence of stroke have been reported to increase year by year, with high rates of disability and mortality, and is a major cause of death and disability in the world’s population, seriously jeopardizing human life and health [2].

Currently, thrombolysis applied to achieve early reperfusion is the most valid method of treating acute cerebral ischemia. Still, this therapy is limited by the time window of thrombolysis, and successful thrombolysis causes substantial damage to brain tissue with cerebral ischemia (ischemia/reperfusion injury (I/R)), which may be related to inflammatory responses, mitochondrial dysfunction, increased production of ROS, oxidative stress, and activation of cell death pathways [3–8]. For many years, despite many studies in this field, we did not significantly improve the prognosis and treatment of ischemic stroke patients. Therefore, exploring new targets and therapies to reduce re-injury in cerebral ischemia remains a pivotal challenge for treating cerebral ischemia.

Ferroptosis was a new mechanism of programmed cell death, a non-apoptotic programmed death pathway reliant on iron ions and ROS [9]. Ferroptosis is characterized by a significant elevation of iron content and accumulation of lipid peroxides, and lead to cell death and neurological damage after stroke. During ischemia/reperfusion injury, the abnormal increase of cerebral microvascular endothelial cell permeability severely damages the blood–brain barrier (BBB), which allows a large amount of iron to enter the brain parenchyma and trigger iron overload. The increased iron content results in the disturbance of cerebral iron metabolism and eventually contributes to the occurrence of cellular ferroptosis [10, 11]. During cerebral ischemia, local cerebral tissue blood supply is insufficient, mitochondrial dysfunction occurs, and ATP can not be produced. After reperfusion of cerebral blood flow, oxidative stress increases mitochondrial damage further [12, 13] and generates excessive ROS [14]. The ROS accumulation-mediated oxidative stress and lipid peroxidation are the key driving forces triggering cellular ferroptosis [15, 16]. In addition, overaccumulation accumulation of extracellular glutamate is also a major cause of neuronal cell death, which exerts oxidative toxicity by inhibiting cystine absorption through restraining the activity of System Xc-. As the light chain of the Xc system, SLC7A11 translocates a molecule of glutamate out of the cell and exchanges a molecule of cystine into the cell in a 1:1 ratio. Cystine is necessary for the synthesis of glutathione (GSH) [17]. Intracellular GSH depletion reduces the activity of GPX4 and further leads to the dysfunction of lipid peroxides metabolism [18]. Excess Fe^2+^ oxidizes lipids employing the Fenton reaction, generating massive amounts of ROS and eventually leading to ferroptosis [19–21]. As shown in many studies, inhibition of ferroptosis is pivotal in rescuing neuronal damage after brain I/R injury.

Electroacupuncture (EA), integrates traditional acupuncture with modern electric stimulation and has been widely used in treating stroke and poststroke with significant clinical effects, and less undesirable effects. EA therapy has been demonstrated to be neuroprotective against ischemic stroke by effectively attenuating a wide range of pathologic processes. In addition, the co-application of EA at the “Baihui” point and the “Zusanli” point had a synergistic protective effect on mitigating neuronal damage in MCAO rats by curbing endoplasmic reticulum stress and ameliorating the defective mitochondrial function [22]. Therefore, EA can effectively restrain the ischemic-hypoxic cascade response and neuronal injury [23]. Recent studies have found that EA intervention in MCAO rats can also repress the formation of ferroptosis, which protects damaged neuronal cells in MCAO rats [24]. However, the mechanism of the way by which EA inhibits ferroptosis is still obscure. Investigating the specific mechanism of EA for ischemic stroke is anticipated to yield a new scientific basis for targeted therapy of relevant neurological diseases in the future.

The etiopathogenesis of ferroptosis is sophisticated and involves multiple transcription factors including p53, NFE2L2, and Nrf2 [25, 26]. Among them, Nrf2, a crucial ferroptosis regulator, is known to transcribe several antioxidant response element (ARE)-containing genes to maintain redox homeostasis [27, 28]. Therefore, deficiency of Nrf2 causes increased susceptibility to brain injury [29–31]. Under normal conditions, Nrf2 binds to Kelch-like ECH-associated protein1 (Keap1), an interface protein for Cul3 E3 ubiquitin ligases, which is accountable for the ubiquitination and degradation of Nrf2 [32, 33]; under oxidative stress, Nrf2 dissociates from Keap1, translocates to the nucleus and activates the transcription of the ARE. The ARE transcribes and drives a variety of antioxidant genes, including heme oxygenase-1 (HO-1), to exert antioxidant effects [34–38]. As a transcription factor, Nrf2 can directly regulate the expression of several important genes in the process of ferroptosis in the nucleus, including SLC7A11, GPX4, FTH1, etc. [33, 39, 40], which in turn regulates intracellular iron metabolism, GSH levels, GPX4 synthesis, lipid oxidation, etc. [41, 42]. Therefore, activation of Nrf2 is a valuable target for treating cerebral ischemia [43, 44].

The purpose of this research was to explore the molecular mechanisms regarding the beneficial efficacy of EA in the rat model of MCAO/R-induced brain injury, and it was found that EA could prevent neuronal ferroptosis after ischemic stroke by facilitating the Nrf2 nuclear translocation and activating the Nrf2/SLC7A11/GPX4 pathway, which in turn achieves a neuroprotective effect on the nervous system.

## Materials and methods

### Preparation of MCAO/R rat models and group

Male SPF Sprague–Dawley (SD) rats (200 ± 20 g) were obtained from Hunan Slack (Certificate No.: SCXK (Xiang) 2019–0004) and reared in the Laboratory Animal Center under a 12 h light/dark cycle at 21 ± 2 °C and 60–70% humidity. These rats had unlimited access to food and water. All animal experiments were granted by the Animal Ethics Committee of Yunnan University of Traditional Chinese Medicine (R-062022LH061). The middle cerebral artery occlusion/reperfusion (MCAO/R) model was constructed with the Longa method [45]. In brief, the rats were sterilized by intraperitoneal injection of 3% sodium pentobarbital (40 mg/kg) anesthesia and fastened to an operating platform, and the neck hair was removed, and the muscles and connective tissues were separated. Then the left common carotid artery, external carotid artery, and internal carotid artery. The external carotid artery was tied with a thin line and a minor incision was cut in the external carotid artery. A nylon monofilament (Beijing Sinon Technology Co., Ltd., Beijing, China) with a silicone coating at the tip was then plunged into the internal carotid artery through a tiny incision to a depth of approximately 18–20 mm at the bifurcation. During ischemia, these rats were placed on an insulating pad at 37 °C for 60 min. 60 min later, the nylon monofilament was withdrawn to establish reperfusion and the wound was sutured. Sham rats underwent all steps apart from insertion and extraction of the nylon monofilament.

After 24 h of surgery, the success of MCAO/R was appraised with a neurobehavioral score. 72 rats with successful MCAO/R were randomly numbered and categorized into four groups (*n* = 18): the MCAO/R group, the EA + MCAO/R group, the EA + MCAO/R + DMSO group, and the EA + MCAO/R + Brusatol group, the Sham group was used for control (*n* = 18).

### Electroacupuncture intervention

EA intervention on Quchi (LI11), Baihui (GV20), Dazhui (GV14), and Neiguan (P6) was performed in the MCAO/R + EA group, the EA + MCAO/R + DMSO group, and the EA + MCAO/R + Brusatol group at 24 h after modeling [46]. The Baihui point is located at the top of the head, where the Mai qi converges. Stimulating the Baihui point can directly or indirectly connect with the meridian system of the whole body, thus achieving the effect of nourishing the blood. The Dazhui point belongs to the Governor's Chakra, which has the effect of relieving spasms, relaxing contracture, calming the mind, and strengthening the body; Quchi point applies to all clinical disciplines and is particularly good in Chinese medicine internal diseases, among which paralysis in limb meridian diseases is the most specialized; Neiguan point has the effect of enlightening the mind and tranquilizing the mood, so Neiguan point can treat the diseases of the mind. Therefore, these four acupoints we have chosen are indicated for ischemic stroke treatment. The specific parameters of acupoint were as follows: Baihui point: located in the middle of the parietal bone, and obliquely stabbed forward for 2 mm; Dazhui point: located in the posterior midline and depression below the spinous process of the seventh cervical vertebrae, and stabbed straightly for 5 mm; the right Quchi point: located in the depression in the proximal end of the radius just anterior to the lateral side of the elbow joint, and stabbed straightly for 4 mm; and the right Neiguan point: the medial side of the forelimb, the About 3 mm from the wrist joint, between the ulnar-radial suture, straight stabbing 1 mm. The disposable acupuncture needle (Beijing Zhongyan Taihe Medical Instrument Co., Ltd., Beijing, China) was used for stabbing and then connected to the EA instrument (Changzhou Indy Electronic Medical Instrument Co., Ltd., Changzhou, China), with a current of 2 mA, the frequency of 2 Hz, sparse and dense waves, 30 min/time, 1 time/day for consecutive 7 days [47, 48].

Rats in the EA + MCAO/R + Brusatol group were injected intraperitoneally with the Nrf2 inhibitor Brusatol ((2 mg/kg; Shanghai Yuanye Bio-Technology, CAS number: 14907–98-3, (Shanghai, China)) [49] at 30 min before EA treatment on the first day after modeling, and every other day until the seventh day.

### Neurological severity scores

On the seventh day after MCAO/R, all rats were scored for neurologic severity. Neurological scoring was performed by the same investigator in a blinded manner, and the rats were rated for neurological deficits using a 7-point NSS scale. Higher NSS indicated poorer neurological function [50].

### Garcia score

All rats were blinded by the same researcher, and the Garcia score was based on six main characteristics: voluntary locomotion, body symmetry, forelimb extension, grasp, and climb ability, tactile reflexes on both sides of the body, and whisker touch response on both sides of the body. The total score is 18, with higher scores meaning less nerve impairment [51].

### Foot-fault test

Motor coordination in rats was evaluated by the foot fault test. The test was performed three times consecutively at 5-min intervals. The experiment was scored by the state of the rat's right forefoot grasping the horizontal ladder each time [52], and higher scores represented the better walking ability of the rats.

### Rotarod test

The Rotarod test can assess the recovery of motor function and tolerance level of rats, the operation is as follows: the rats were placed on the rotating rod, the velocity of which was raised from 0 to 10 rpm in 10 s for 5 min. The test was performed three times, recording the time of the rats running on the rotating rod, and average values were taken for statistical analysis [53].

### TTC staining

On postoperative day 7, rats were dosed intraperitoneally with 3% sodium pentobarbital, and the brains were immediately removed and refrigerated for 5 min at −20 °C. The brains were then sliced into 2 mm thick sections. There were 6 slices in total, which were put into a Petri dish containing 2% TTC staining solution (Solarbio Company, Beijing, China), and then covered with tin foil for light protection, and then put into a 37 ℃ incubator to incubate for 15 min, and then brain slices were taken out and arranged in an anterior–posterior order, photographs were taken of these sections. The pale areas were characterized as infarcted. Infarct volume was counted using Image J software (Media Cybernetics, Rockville, MD, USA). Percentage of infarct volume (%) = (infarct volume/total volume) × 100%.

### Nissl staining

Rats were perfused transcardially with 0.9% NaCl and 4% paraformaldehyde solution. The brains were taken out and fixated with paraformaldehyde solution for 24 h before being immersed in 30% sucrose solution at 4 °C for 24 h. After removal, the brains were embedded in paraffin and cut into 4 μm thick slices on a paraffin slicer in preparation for Nissl staining. Briefly, slices of each group were degreased in graded alcohol (70%, 95%, and 100% alcohol) for 3 min, followed by hydration in graded alcohol (95%, 70%, and 50% alcohol) for 3 min. Next, the slices were immersed in a toluidine blue solution at 50–60 °C for 40 min. Following washing with distilled water, the slices were dehydrated once in 70%, 80%, and 95% ethanol for a total of 3 min each, and then twice in 100% ethanol solution. Finally, the slices were immersed in 100% dimethylbenzene solution for 5 min and then sealed with a drop of neutral resin.

### Iron content

Fe^2+^ levels in brain tissue were measured by use of an iron content assay kit (ab83366, Abcam). Measure the absorbance at a wavelength of 593 nm with a spectrophotometer based on the manufacturer's instructions.

### ROS, SOD, and MDA assays

ROS levels were assayed by a ROS kit (E004-1-1, Nanjing Jianjian Biotechnology Co., Ltd.) according to the manufacturer's instructions. Fluorescence intensity was measured with a luciferase marker (Spectra Max Gemini EM, Molecular Devices, USA) with 485 nm excitation wavelength and 530 nm emission wavelength. The MDA assay kit (Cat#BC0025, Solarbio) and SOD assay kit (Cat #BC0170, Solarbio) were performed to detect MDA and SOD levels.

### Extraction of nuclear and cytoplasmic proteins

Nucleoprotein and cytoplasmic proteins were isolated using the Nucleoprotein Extraction Kit (ab113474, Abcam) following the manufacturer's instructions. After obtaining nuclear and cytoplasmic proteins, Nrf2 was quantified in the nucleus and cytoplasm by Western blotting and Lamin-B1 was applied as an up-loading control for nuclear proteins. Data were resolved by Image J.

### Western blot

Rat brain samples were cleaved with RIPA lysis solution (Solarbio, Beijing, China) containing PMSF and phosphatase inhibitors. Total proteins were collected and quantified by BCA protein assay kit (Biosharp, Beijing, China). Total proteins were detached in SDS-PAGE gels and shifted to PVDF (Merck, USA) membranes. The membranes were occluded with 5% skimmed milk (Biofroxx) for 2 h at room temperature and then incubated with primary antibodies at 4 °C overnight. Primary antibodies included Anti-Nrf2 (1:1000; ab137550; Abcam), Anti-pNrf2 (1:1000; ab76026; Abcam), Anti-GPX4 (1:1000; ab125066; Abcam), Anti-HO-1 (1:1000; ab13243; Abcam), Anti-FTH1 (1:1000; ab65080; Abcam), Anti-SLC7A11 (1:1000; ab175186; Abcam), Anti-β-actin (1:1000; 3700; Cell Signaling Technology, USA), and Anti-Lamin-B1 (1:1000; ab16048, Abcam). The next day, the membranes were cleaned with TBST three times, and then incubated with HRP-coupled goat anti-rabbit IgG secondary antibody (1:5000; S0001; Affinity Biosciences) at room temperature for 1 h. After three washings, the membranes were visualized on a chemiluminescence detection system (Clink Science Instruments Co., Ltd., Shanghai, China) with ECL chemiluminescence reagent (Biosharp Life Science Co., Ltd.) and analyzed with Image J software.

### Immunofluorescence

Rats were anesthetized intensively and perfused transcardially with 0.9% NaCl and 4% paraformaldehyde. Whole brains were removed and immobilized in 4% paraformaldehyde through the night, and then dehydrated with a sucrose gradient at room temperature. The brains were encapsulated in OCT and sectioned into 10 μm thick slices using a cryosectioner. Sections were incubated with 5% bovine serum albumin (BSA) blocked at room temperature for 1 h and then hatched overnight at 4 °C with Anti-GPX4 (1:100; ab125066; Abcam), Anti-Nrf2 (1:100; ab137550; Abcam), and Anti-NeuN (1:200; ab104224; Abcam). Subsequently, sections were rinsed three times with PBS and incubated with a fluorescent secondary antibody (Alexa Fluor 488/594 AffiniPure Goat Anti-Rabbit IgG (H + L); Jackson ImmunoResearch) and then in a 37 °C incubator. The sections were then incubated with DAPI for 5 min at room temperature. Positive signals in the ischemic penumbra were visualized and photographed using a Zeiss LSM-710 confocal microscope. Fluorescence intensity was analyzed using ImageJ software.

### Transmission electron microscopy

Brain tissues of 1 × 1 × 2 mm size from the ischemic penumbra were stabilized in 3% glutaraldehyde solution for 1 h and then in 1% osmium tetroxide solution for 2 h. The sections were then dehydrated in acetone and finally imbedded in Epon812. Ultrathin sections of 60–90 nm were fabricated using an ultrathin sectioning machine. The sections were then colored with uranyl acetate for 10–15 min at room temperature, then with lead citrate for 1–2 min, and lastly, the morphology of mitochondria was investigated by transmission electron microscopy (JEM-1400FLASH, Japan).

### Statistical analysis

Data were statistically analyzed using SPSS 26.0 software. All experimental data were reported as (mean ± SD). One-way ANOVA with a 95% confidence interval was used to assess the differences between three or more experimental groups. Tukey’s multiple comparison post-hoc test was then performed, *p* < 0.05 denoted a statistically significant difference. Graphs were plotted using GraphPad Prism 9.5 software.

## Results

### Neuroprotective effect of EA against cerebral MCAO/R induced injuries

**Fig. 1:**
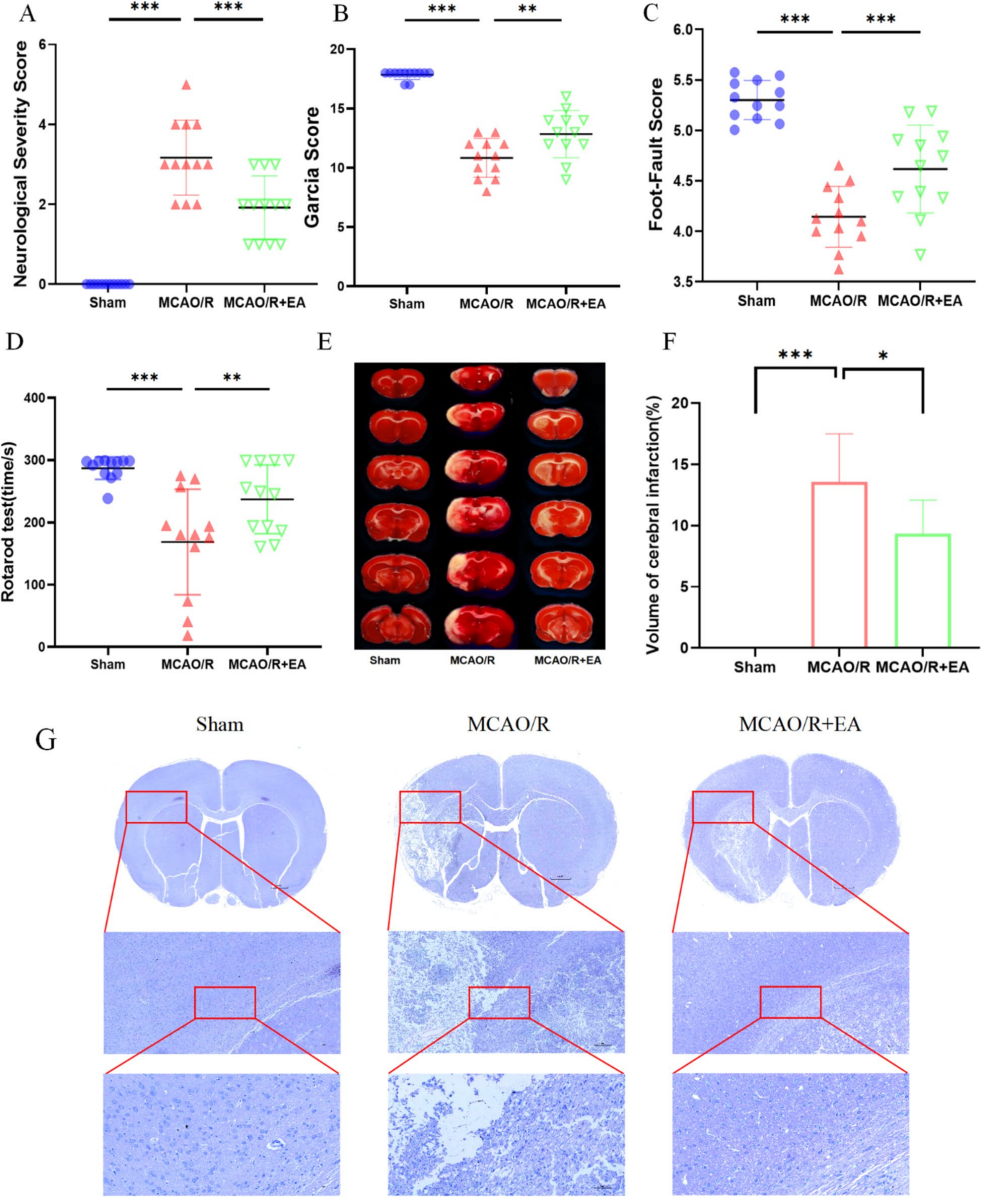
Neuroprotective effect of EA against cerebral MCAO/R induced injuries. **A**–**D** Neurological deficits of each group were scored with the NSS, Garcia score, Foot-fault test, and Rotarod test at 7 days after surgery (*n* = 12). **E**, **F** Representative images of TTC stained section and quantitation (*n* = 6). **G** Representative photomicrographs of Nissl stained sections (Scale bar: 1 mM, 200 μM, and 50 μM, *n* = 6). *p < 0.05, **p < 0.01, ***p < 0.001

The neurofunctional recovery was measured by NSS and Garcia score on days 7 after MCAO/R. The results indicate that the neurological function of rats in the MCAO/R group was markedly corrupted. EA markedly improved the neurological deficits. The NSS in the MCAO/R group was significantly higher than that in the Sham group, EA treatment significantly improved the neurological deficits compared with the MCAO/R groups (2.83 ± 0.72 in the MCAO/R group vs. 0.00 ± 0.00 in the Sham group, *p* < 0.001; 1.92 ± 0.79 in the MCAO/R + EA group vs. 2.83 ± 0.72 in the MCAO/R group, *p* < 0.001; Fig. 1A). Similarly, the Garcia score was significantly lower in the MCAO/R group than in the Sham group, and the EA treatment improved the Garcia score (10.83 ± 1.64 in the MCAO/R group vs. 17.83 ± 0.39 in the Sham group, *p* < 0.001; 12.83 ± 1.99 in the MCAO/R + EA group vs. 10.83 ± 1.64 in the MCAO/R group, *p* < 0.01; Fig. 1B). The results of the Foot-fault Test (4.14 ± 0.30 in the MCAO/R group vs. 5.30 ± 0.19 in the Sham group, *p* < 0.001; 4.62 ± 0.44 in the MCAO/R + EA group vs. 4.14 ± 0.30 in the MCAO/R group, *p* < 0.001; Fig. 1C) and the Rotarod Test (168.47 ± 84.73 in the MCAO/R group vs. 286.97 ± 18.03 in the Sham group, *p* < 0.001; 236.92 ± 55.13 in the MCAO/R + EA group vs. 168.47 ± 84.73 in the MCAO/R group, *p* < 0.01; Fig. 1D) revealed that exercise capacity was enhanced in the MCAO/R + EA group as compared to the MCAO/R group. Major cerebral infarcts were noticed in MCAO/R operated rats by TTC staining compared to the Sham group. Yet, the volume of cerebral infarction was diminished in the EA + MCAO/R group compared with the MCAO/R group. (Fig. 1E; 13.58 ± 3.91 in the MCAO/R group vs. 0.00 ± 0.00 in the Sham group, *p* < 0.001; 9.34 ± 2.74 in the MCAO/R + EA group vs. 13.58 ± 3.91 in the MCAO/R group, *p* < 0.05; Fig. 1F). Meanwhile, Nissl staining indicated that compared with the Sham group, the neuronal cells in the MCAO/R group were disorganized or missing, and the Nissl body staining was incomplete or even dissolved, suggesting that neuronal damage was induced by MCAO/R modeling. The infarct volume was decreased and the neuronal cell recovery in the ischemic rats with EA treatment compared with that of the ischemic rats without treatment (Fig. 1G).

### EA attenuates MCAO/R-induced ferroptosis

**Fig. 2:**
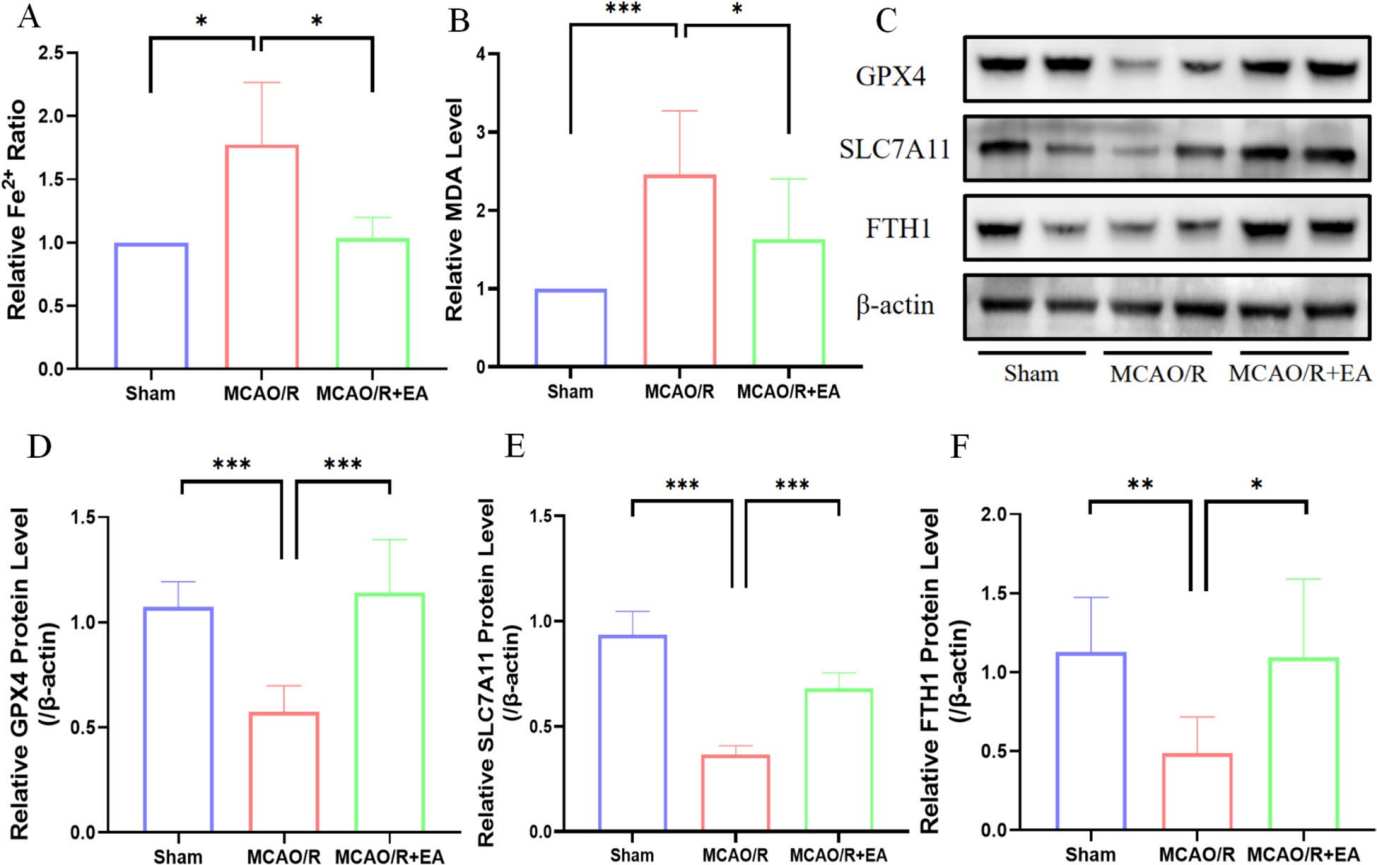
EA inhibits ferroptosis in rats following MCAO/R. **A**, **B** The concentration of Fe^2+^, and MDA in brain tissue (*n* = 6). **C** The representative Western blotting results of GPX4, SLC7A11, and FTH1 expression. **D**–**F** Quantification of GPX4, SLC7A11, and FTH1 expressions (*n* = 6). *p < 0.05, **p < 0.01, ***p < 0.001

MDA and Fe^2+^ levels were elevated distinctly in the MCAO/R and EA + MCAO/R groups relative to the Sham group. Yet, the levels of MDA (1.78 ± 0.49 in the MCAO/R group vs. 1.00 ± 0.00 in the Sham group, *p* < 0.05; 1.03 ± 0.16 in the MCAO/R + EA group vs. 1.78 ± 0.49 in the MCAO/R group, *p* < 0.05; Fig. 2A) and Fe^2+^ (2.46 ± 0.82 in the MCAO/R group vs. 1.00 ± 0.00 in the Sham group, *p* < 0.001; 1.63 ± 0.77 in the MCAO/R + EA group vs. 2.46 ± 0.82 in the MCAO/R group, *p* < 0.05; Fig. 2B) in the EA + MCAO/R group were decreased compared with those in the MCAO/R group. Similarly, the expression of ferroptosis-related proteins, GPX4 (Fig. 2C; 0.58 ± 0.13 in the MCAO/R group vs. 1.07 ± 0.12 in the Sham group, *p* < 0.001; 1.14 ± 0.25 in the MCAO/R + EA group vs. 0.58 ± 0.13 in the MCAO/R group, *p* < 0.001; Fig. 2D), SLC7A11 (0.37 ± 0.04 in the MCAO/R group vs. 0.94 ± 0.11 in the Sham group, *p* < 0.001; 0.68 ± 0.07 in the MCAO/R + EA group vs. 0.37 ± 0.04 in the MCAO/R group, *p* < 0.001; Fig. 2E), and FTH1 (0.49 ± 0.23 in the MCAO/R group vs. 1.13 ± 0.35 in the Sham group, *p* < 0.01; 1.09 ± 0.50 in the MCAO/R + EA group vs. 0.49 ± 0.23 in the MCAO/R group, *p* < 0.05; Fig. 2F) was reduced substantially in the MCAO/R group despite the Sham group; nonetheless, the EA intervention counteracted this trend in the EA + MCAO/R group.

**Fig. 3:**
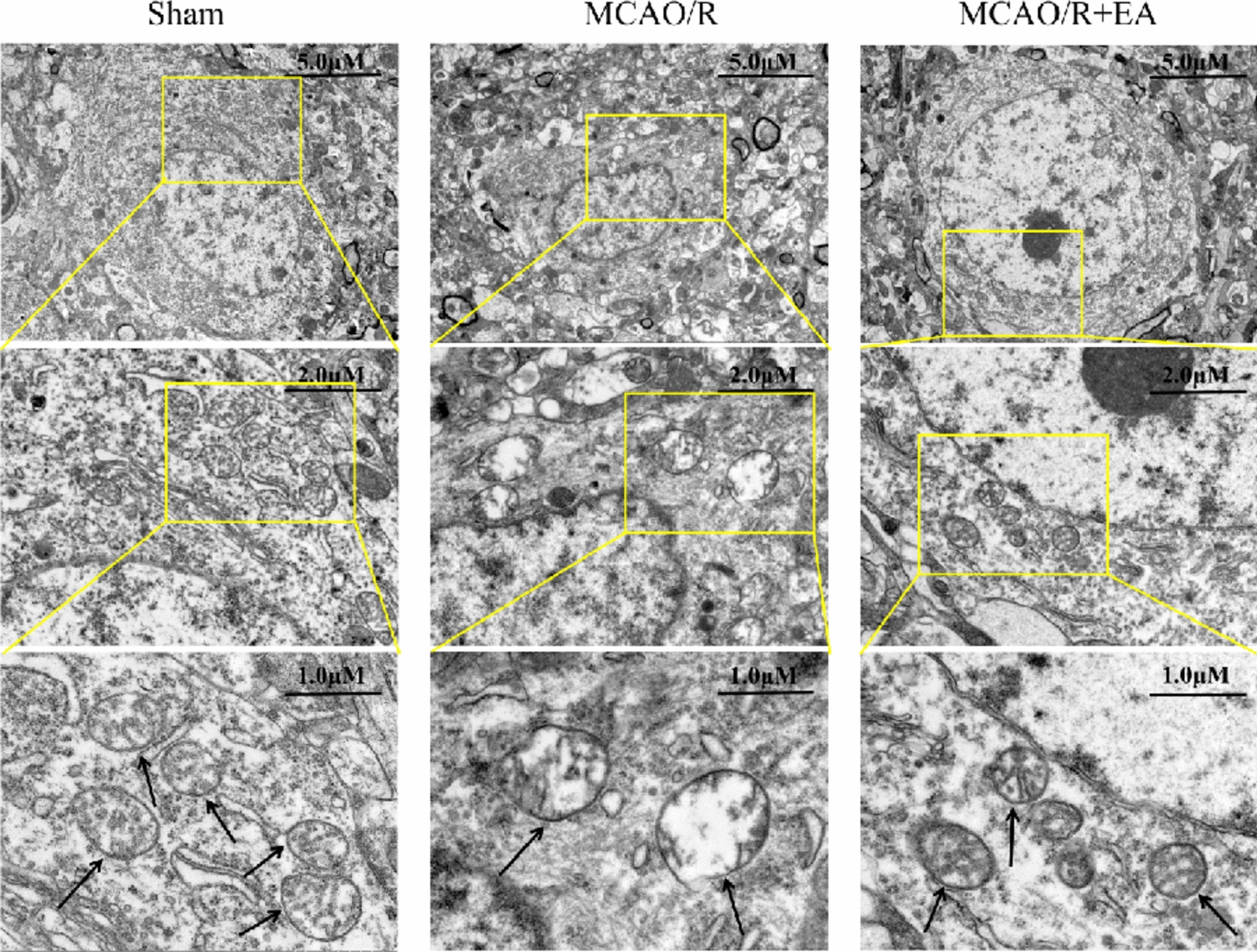
EA inhibits ferroptosis in rats following MCAO/R. The bottom panels display the magnified images of regions indicated by yellow rectangles in the top panels. Arrows labeled: representative images of mitochondria. (Scale bar: 5 μM, 2 μM, and 1 μM, *n* = 3)

Ferroptosis damages the structure and functional impairment in mitochondria. We observed the mitochondrial ultrastructure by TEM. The samples exhibited irregular mitochondrial morphology, proliferation of vacuoles, and invagination of the mitochondrial membrane after MCAO/R, whereas the mitochondrial morphology in the MCAO/R + EA group was standardized with fewer vacuoles. (Fig. 3).

### EA increases the expression of Nrf2, and promotes Nrf2 phosphorylation and nuclear translocation

**Fig. 4:**
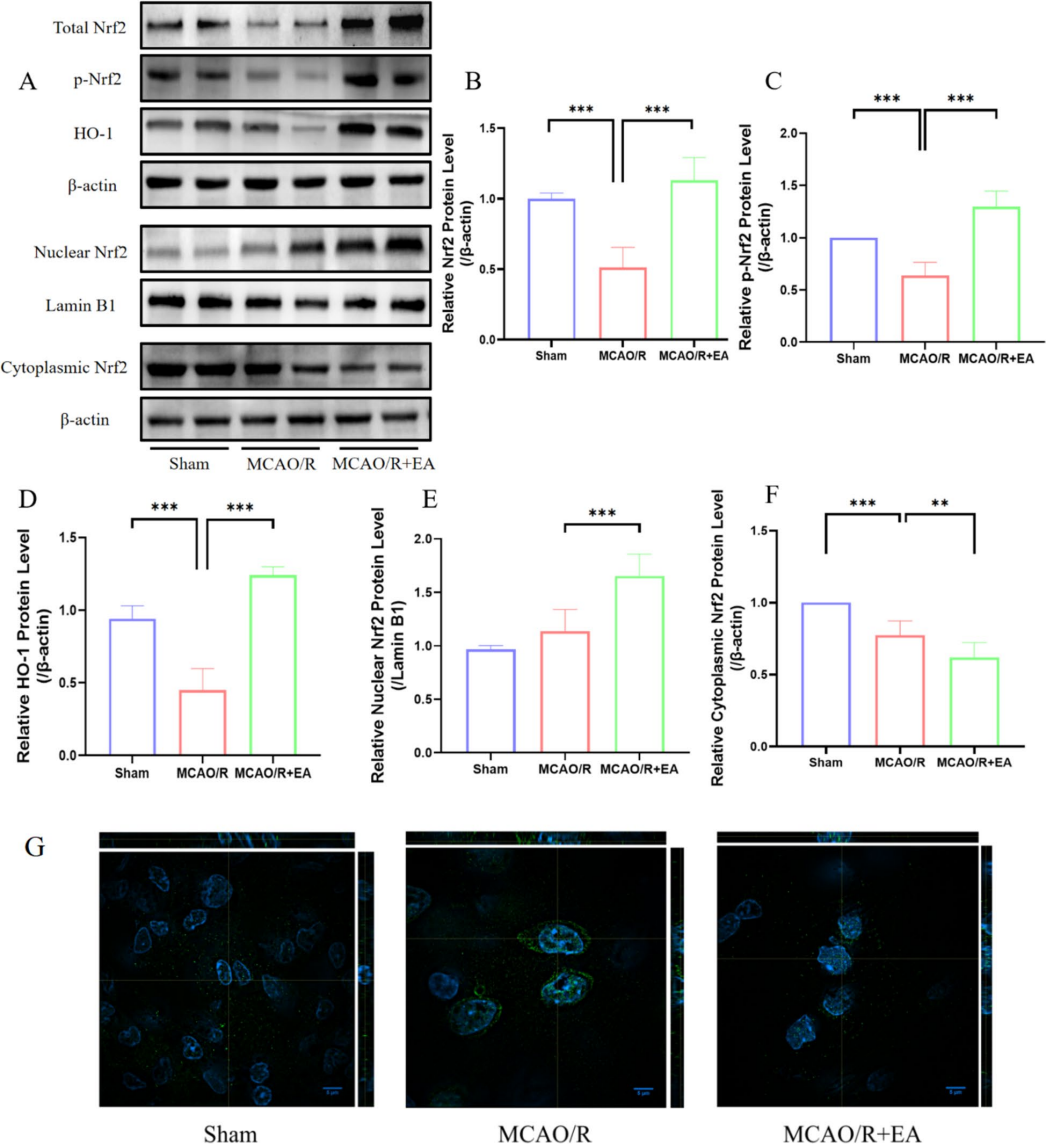
EA increases the expression of Nrf2 and promotes Nrf2 phosphorylation and nuclear translocation. **A** The representative Western blotting bands of Total Nrf2, p-Nrf2, HO-1, Nuclear Nrf2, and Cytoplasmic Nrf2 expression. **B**–**F** Quantification of Total Nrf2, p-Nrf2, HO-1, Nuclear Nrf2 and Cytoplasmic Nrf2 expressions (*n* = 6). *p < 0.05, **p < 0.01, ***p < 0.001. **G** The confocal images of Nrf2. Scale bar: 5 μm. Nrf2 and DAPI were excited at 488 and 405 nm, respectively. Exemplary XY plane of confocal z-stack acquisition of Nrf2 (green signal) and treated with DAPI for nuclear staining (blue signal). Orthogonal views (XZ and YZ planes) extracted from z-stack are reported

To probe the molecular mechanism by which EA anti-ferroptosis potential, a crucial factor of the Nrf2 pathway was determined by the researchers. The outcomes revealed that the expression and phosphorylation values of Nrf2 and HO-1 were pronouncedly under-regulated in the MCAO/R group. However, the decrease of total Nrf2 (Fig. 4A; 0.51 ± 0.14 in the MCAO/R group vs. 1.00 ± 0.04 in the Sham group, *p* < 0.001; 1.13 ± 0.16 in the MCAO/R + EA group vs. 0.51 ± 0.14 in the MCAO/R group, *p* < 0.001; Fig. 4B), p-Nrf2 (0.64 ± 0.13 in the MCAO/R group vs. 1.00 ± 0.00 in the Sham group, *p* < 0.001; 1.30 ± 0.15 in the MCAO/R + EA group vs. 0.64 ± 0.13 in the MCAO/R group, *p* < 0.001; Fig. 4C) and HO-1 (0.45 ± 0.15 in the MCAO/R group vs. 0.94 ± 0.09 in the Sham group, *p* < 0.001; 1.24 ± 0.06 in the MCAO/R + EA group vs. 0.45 ± 0.15 in the MCAO/R group, *p* < 0.001; Fig. 4D) was rescued by EA treatment. Intriguingly, nuclear Nrf2 was slightly elevated in the MCAO/R group and markedly elevated in the EA + MCAO/R group, whereas cytoplasmic Nrf2 was strikingly diminished in both the MCAO/R group and the EA + MCAO/R group. Compared to the MCAO/R group, the cytoplasmic Nrf2 (1.14 ± 0.20 in the MCAO/R group vs. 0.97 ± 0.04 in the Sham group, *p* > 0.05; 1.65 ± 0.20 in the MCAO/R + EA group vs. 1.14 ± 0.20 in the MCAO/R group, *p* < 0.001; Fig. 4E) was less while the nuclear Nrf2 (0.77 ± 0.10 in the MCAO/R group vs. 1.00 ± 0.00 in the Sham group, *p* < 0.001; 0.62 ± 0.10 in the MCAO/R + EA group vs. 0.77 ± 0.10 in the MCAO/R group, *p* < 0.01; Fig. 4F) was more. We further observed the localization of Nrf2 by confocal. Our findings confirmed that Nrf2 mainly accumulates in the cytoplasm in the MCAO/R group, and EA efficiently promotes the nuclear translocation of Nrf2 (Fig. 4G). These results implied that EA promoted the phosphorylation of Nrf2 and nuclear translocation.

### Brusatol reduces the content of Nrf2, inhibits Nrf2 phosphorylation and nuclear translocation

**Fig. 5:**
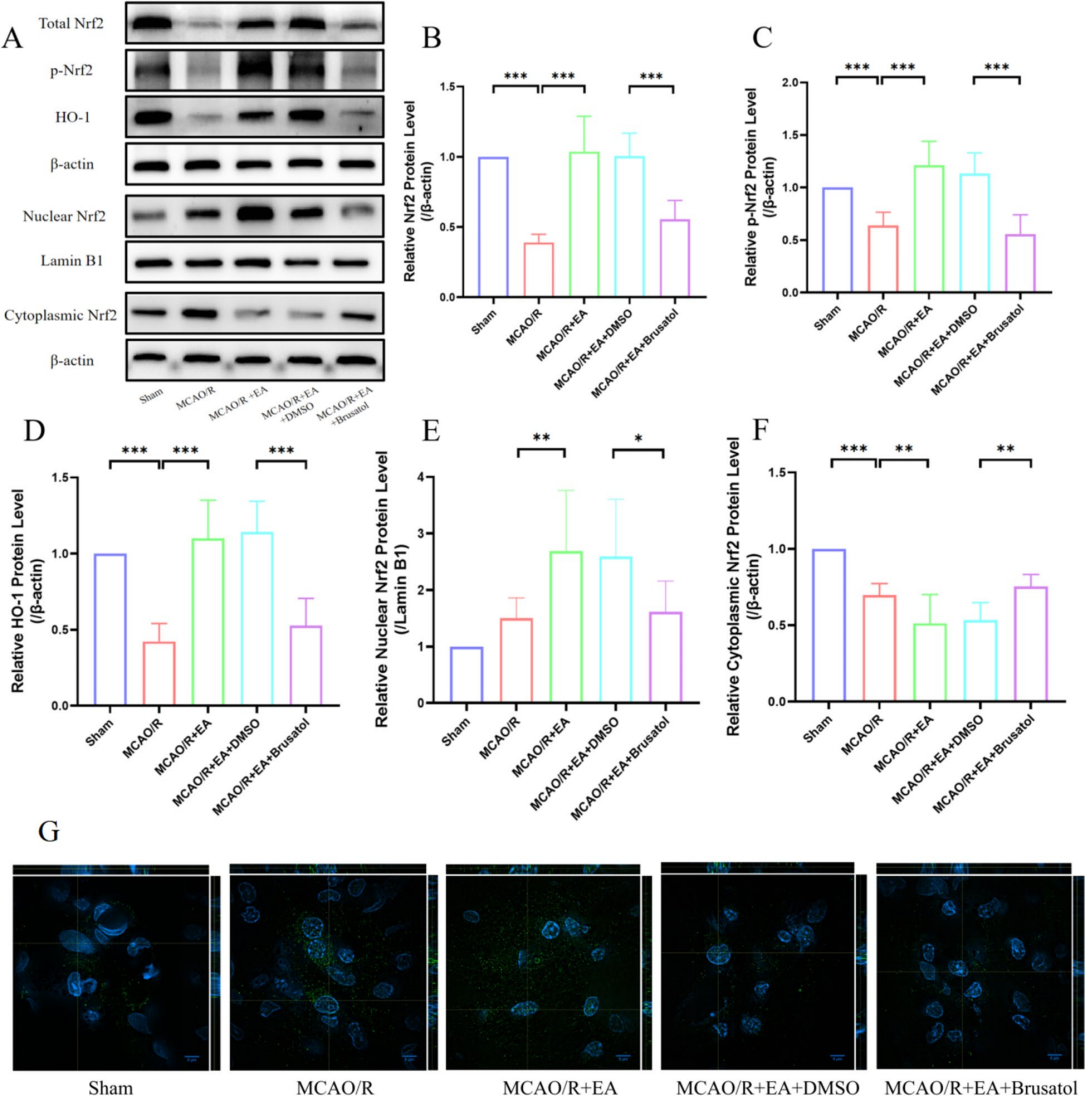
Brusatol reduces the content of Nrf2 and inhibits Nrf2 phosphorylation and nuclear translocation. **A** The representative Western blotting results of Total Nrf2, p-Nrf2, HO-1, Nuclear Nrf2, and Cytoplasmic Nrf2 expression. **B**–**F** Quantification of Total Nrf2, p-Nrf2, HO-1, Nuclear Nrf2 and Cytoplasmic Nrf2 expressions (*n* = 6). *p < 0.05, **p < 0.01, ***p < 0.001. **G** The confocal images were performed to determine the protein expressions of Nrf2. Scale bar: 5 μm. Nrf2 and DAPI were excited at 488 and 405 nm, respectively. Exemplary XY plane of confocal z-stack acquisition of Nrf2 (green signal) and treated with DAPI for nuclear staining (blue signal). Orthogonal views (XZ and YZ planes) extracted from z-stack are reported

To further verify the effects of Nrf2 in MCAO/R-induced ferroptosis, we inhibited the Nrf2 by brusatol. The expression of Total Nrf2 (Fig. 5A; 1.01 ± 0.16 in the MCAO/R + EA + DMSO group vs. 0.56 ± 0.13 in the MCAO/R + EA + Brusatol group, *p* < 0.001; Fig. 5B), p-Nrf2 (1.14 ± 0.20 in the MCAO/R + EA + DMSO group vs. 0.56 ± 0.18 in the MCAO/R + EA + Brusatol group, *p* < 0.001; Fig. 5C), HO-1 (1.14 ± 0.20 in the MCAO/R + EA + DMSO group vs. 0.53 ± 0.18 in the MCAO/R + EA + Brusatol group, *p* < 0.001; Fig. 5D), Nuclear Nrf2 (2.59 ± 1.01 in the MCAO/R + EA + DMSO group vs. 1.62 ± 0.54 MCAO/R + EA + Brusatol group, *p* < 0.05; Fig. 5E), and Cytoplasmic Nrf2 (0.54 ± 0.11 in the MCAO/R + EA + DMSO group vs. 0.76 ± 0.08 in the MCAO/R + EA + Brusatol group, *p* < 0.01; Fig. 5F) were examined by Western blotting, and the results indicated that brusatol reversed the effects of increased expression and promoted nuclear translocation induced by EA treatment. We further observed the location of Nrf2 by immunofluorescence (Fig. 5G). The results also revealed that EA treatment enhanced the content of Nrf2 and attracted the nuclear translocation of Nrf2 in the MCAO/R + EA group, while Brusatol suppressed the nuclear translocation of Nrf2 prominently. These observations confirm that Brusatol prominently suppressed the total content of Nrf2 and HO-1, and suppressed the phosphorylation and nuclear translocation of Nrf2 upon EA treatment.

### Brusatol diminishes the attenuated ferroptosis induced by EA after MCAO/R

**Fig. 6:**
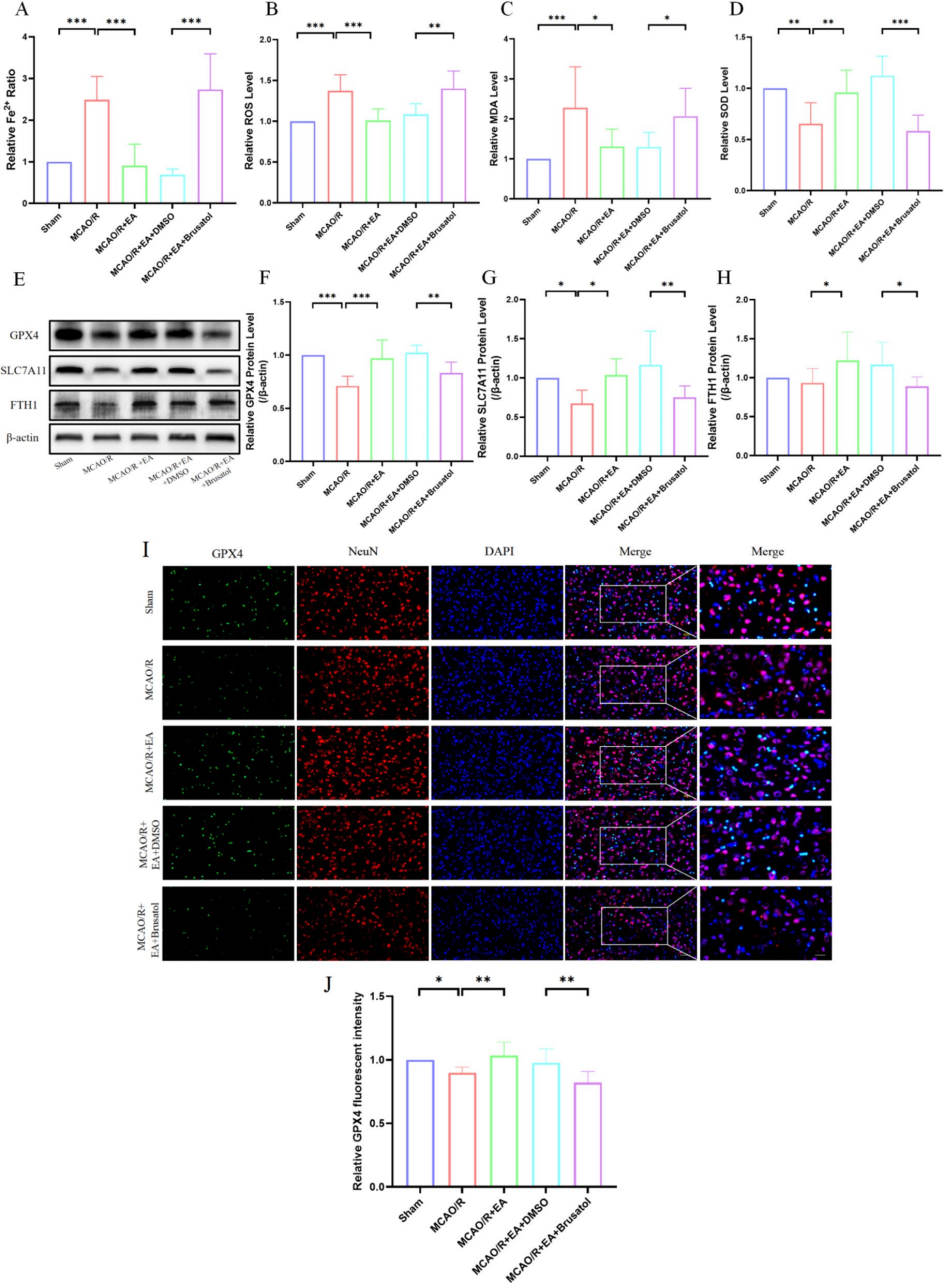
Brusatol diminishes the protective effect of EA that attenuates ferroptosis after MCAO/R. **A**–**D** The concentration of Fe^2+^, ROS, MDA, and SOD in each group's brain tissue (*n* = 6). **E** The representative Western blotting results of GPX4, SLC7A11, and FTH1 expression. (F–H) Quantification of GPX4, SLC7A11, and FTH1 expressions (*n* = 6). **I**, **J** Typical double immunofluorescence images (GPX4 (green), NeuN (red), DAPI (blue). Scale bar: 25 µm. *n* = 6). *p < 0.05, **p < 0.01, ***p < 0.001

Compared with the MCAO/R + EA + DMSO group, the Fe^2+^ content(0.69 ± 0.13 in the MCAO/R + EA + DMSO group vs. 2.74 ± 0.85 in the MCAO/R + EA + Brusatol group, *p* < 0.001; Fig. 6A), ROS (1.08 ± 0.13 in the MCAO/R + EA + DMSO group vs. 1.40 ± 0.22 in the MCAO/R + EA + Brusatol group, *p* < 0.01; Fig. 6B) and MDA levels (1.30 ± 0.36 in the MCAO/R + EA + DMSO group vs. 2.07 ± 0.70 in the MCAO/R + EA + Brusatol group, *p* < 0.05; Fig. 6C) in MCAO/R + EA + Brusatol group were significantly higher, while, the SOD level (1.12 ± 0.19 in the MCAO/R + EA + DMSO group vs. 0.58 ± 0.15 in the MCAO/R + EA + Brusatol group, *p* < 0.001; Fig. 6D) in was significantly lower. Similarly, the results by Western blotting showed that Brusatol significantly down-regulated the expression of GPX4 (Fig. 6E; 1.03 ± 0.07 in the MCAO/R + EA + DMSO group vs. 0.83 ± 0.10 in the MCAO/R + EA + Brusatol group, *p* < 0.01; Fig. 6F), SLC7A11 (1.17 ± 0.43 in the MCAO/R + EA + DMSO group vs. 0.75 ± 0.15 in the MCAO/R + EA + Brusatol group, *p* < 0.01; Fig. 6G) and FTH1 proteins (1.17 ± 0.29 in the MCAO/R + EA + DMSO group vs. 0.89 ± 0.12 in the MCAO/R + EA + Brusatol group, *p* < 0.05; Fig. 6H). The GPX4 expression level was further assessed by Double immunofluorescence staining. The findings displayed that GPX4 was predominantly co-localized with neurons (Fig. 6I). Consistent with GPX4 protein levels, MCAO/R prominently degraded GPX4 expression. EA augmented GPX4 expression, while the Brusatol reversed the up-regulation of GPX4 induced by EA (0.98 ± 0.11 in the MCAO/R + EA + DMSO group vs. 0.82 ± 0.09 in the MCAO/R + EA + Brusatol group, *p* < 0.01; Fig. 6J). These findings demonstrate that brusatol counteracts the protectiveness of EA through the Nrf2 pathway.

**Fig. 7:**
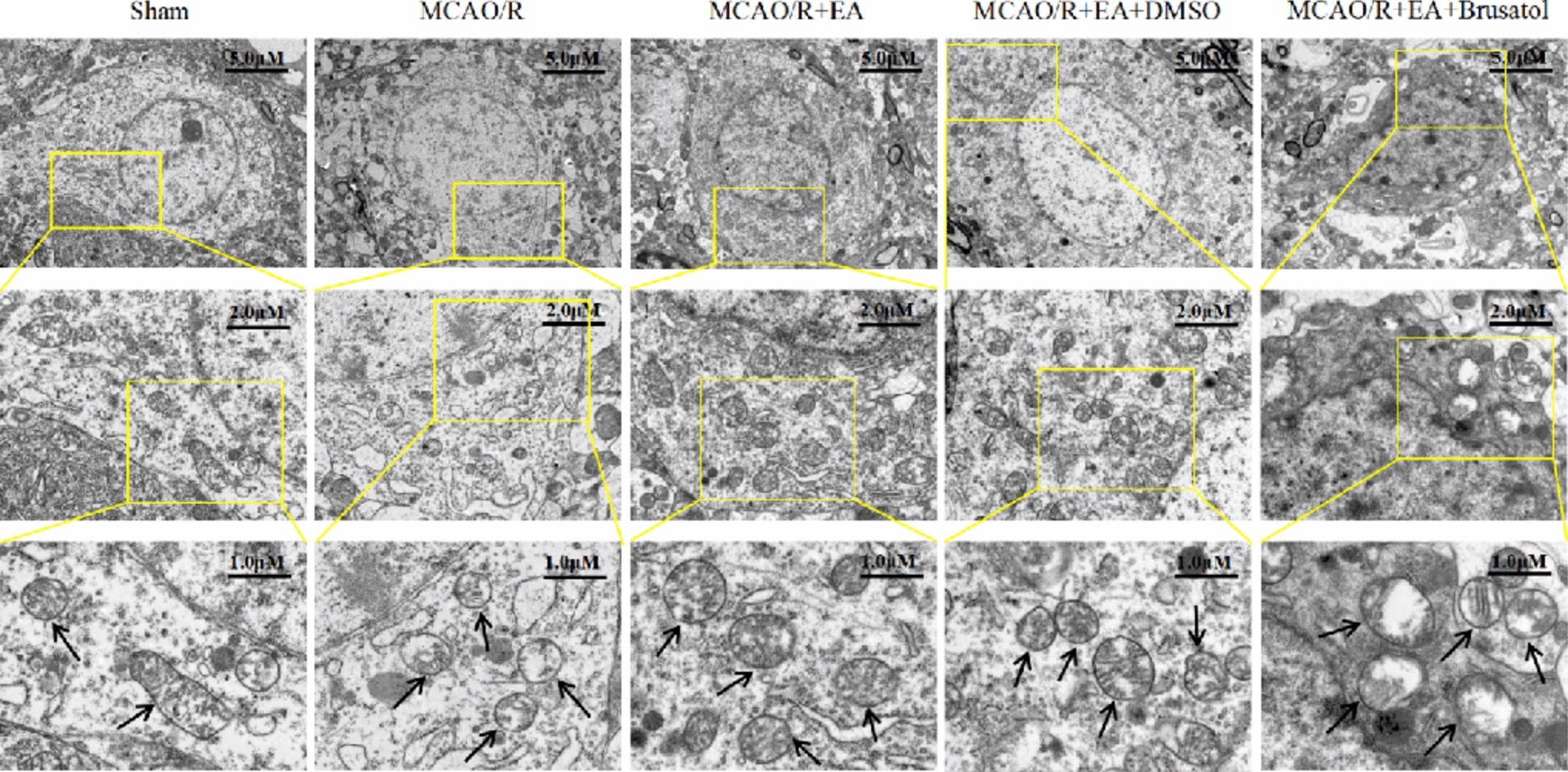
Brusatol diminishes the protective effect of EA that attenuates ferroptosis after MCAO/R. Mitochondrial morphology associated with ferroptosis was determined by TEM. The bottom panels display the magnified images of regions indicated by yellow rectangles in the top panels. Arrows labeled: representative images of mitochondria. (Scale bar: 5 μM, 2 μM, and 1 μM, *n* = 3)

Next, TEM was used to observe the morphology of mitochondria. As shown in the images, mitochondria in the Sham group had an intact outer membrane, abundant cristae, and normal morphology, MCAO/R led to the production of fragmented mitochondria with decreased cristae, even appeared to be vacuolated, which is a characteristic of ferroptosis. However, EA dampened MCAO/R-induced morphological changes in mitochondria (Fig. 7). In contrast, Brusatol inverted the advantageous effects of EA on mitochondria.

### Brusatol reverses the neuroprotective effect of EA

**Fig. 8:**
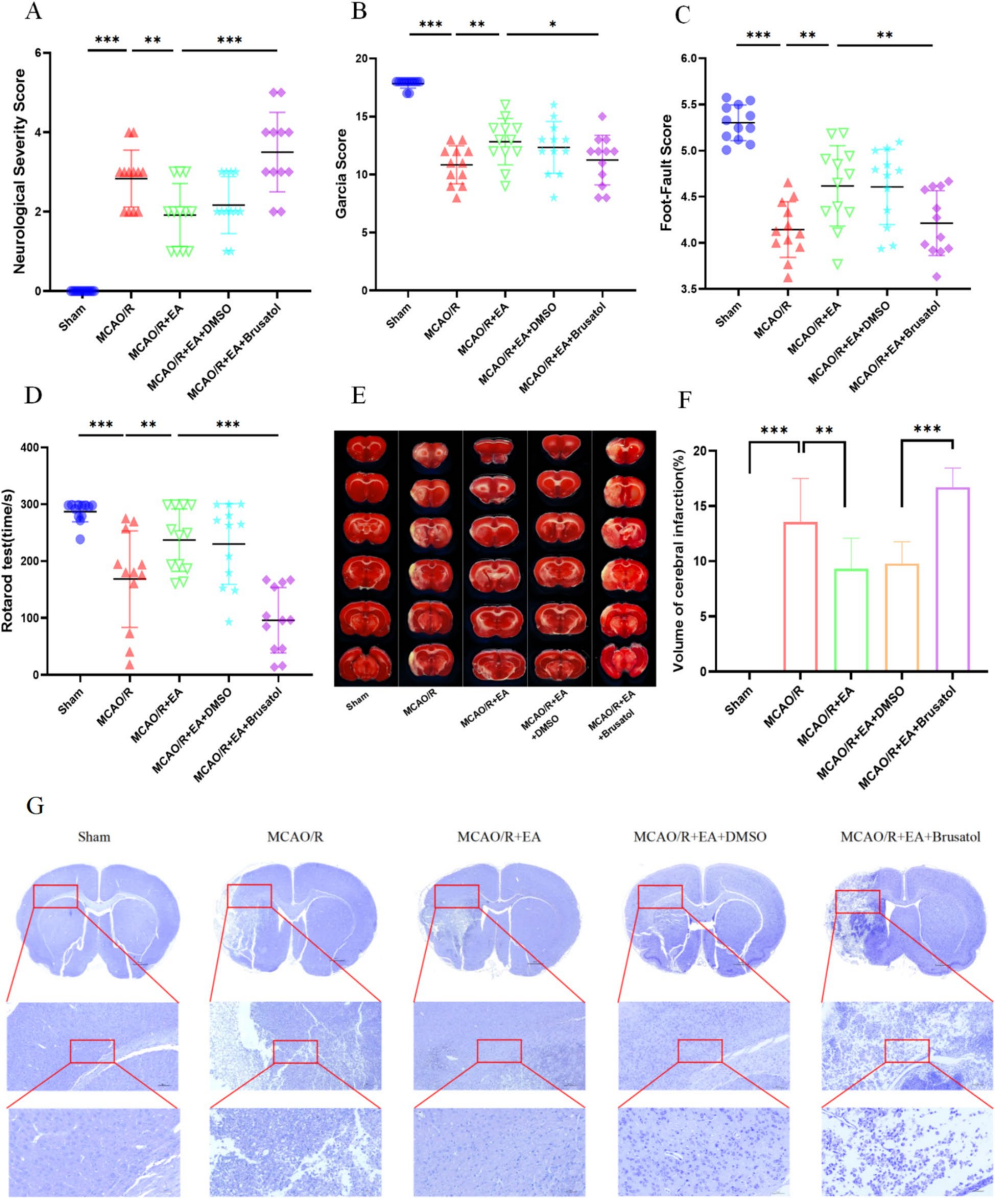
Brusatol reverses the neuroprotective effect of EA. **A**–**D** Neurological deficits of each group were scored with the NSS, Garcia score, Rotarod Test, and Foot-fault Test at 7 days after surgery (*n* = 12). **E**, **F** Evaluation of cerebral infarction volume of rats by TTC staining and analysis (*n* = 6). **G** Representative photomicrographs of Nissl stained sections (Scale bar: 1 mM, 200 μM, and 50 μM, *n* = 6). *p < 0.05, **p < 0.01, ***p < 0.001

Behavioral tests and TTC staining were utilized to appraise the effects of brusatlo on the neuroprotection provided by EA. The results presented that the score of NSS (2.17 ± 0.72 in the MCAO/R + EA + DMSO group vs. 3.50 ± 1.00 in the MCAO/R + EA + Brusatol group, *p* < 0.001; Fig. 8A) was significantly greater and the Garcia score (12.33 ± 2.23 in the MCAO/R + EA + DMSO group vs. 10.75 ± 1.71 in the MCAO/R + EA + Brusatol group, *p* < 0.05; Fig. 8B) was below in the MCAO/R + EA + Brusatol group than in MCAO/R + EA + DMSO group. Similarly, The results of the Foot-fault Test (4.61 ± 0.41 in the MCAO/R + EA + DMSO group vs. 4.21 ± 0.35 in the MCAO/R + EA + Brusatol group, *p* < 0.01; Fig. 8C) and the Rotarod Test (229.97 ± 71.02 in the MCAO/R + EA + DMSO group vs. 96.17 ± 57.38 in the MCAO/R + EA + Brusatol group, *p* < 0.001; Fig. 8D) suggested that functional motor restoration was inferior in the MCAO/R + EA + Brusatol group compared to the MCAO/R + EA + DMSO group. TTC staining revealed that the volume of cerebral infarction in rats in the MCAO/R + EA + Brusatol group was noticeably larger than that in rats in the MCAO/R + EA + DMSO group. (Fig. 8E; 9.79 ± 1.97 in the MCAO/R + EA + DMSO group vs. 16.71 ± 1.74 in the MCAO/R + EA + Brusatol group, *p* < 0.001; Fig. 8F). Meanwhile, Nissl staining results showed that compared with the MCAO/R + EA + DMSO group, the MCAO/R + EA + Brusatol group had an increased volume of cerebral infarction, disorganized neuronal cell arrangement, and more severe brain damage (Fig. 8G). These results suggested that EA provided a neuroprotection against stroke, while the neuroprotective effect was diminished by the inhibition of Nrf2.

## Discussion

In this study, we established the MCAO/R model in rats and treated them with EA for 7 consecutive days starting from one day after modeling, which ultimately diminished infarct volume and improved neurological function and motor behavior scores. The possible neuroprotective mechanism was that EA alleviated MCAO/R-induced ferroptosis by promoting Nrf2 nuclear transposition and activating the Nrf2/SLC7A11/GPX4 pathway.

Ischemic stroke is the prevalent type of stroke with high mortality and restricted functional recovery in survivors [2]. Ischemic stroke occurs with ischemia and hypoxia, which drastically declines the provision of oxygen, glucose, and other nutrients, and blood supply to the brain, consequently leading to disruption of cellular energy metabolism and destruction of neuronal function [54]. The region of infarction formed by a sharp diminution of blood flow to the brain in ischemic stroke is referred to as the ischemic core. Once the local blood flow supply is curtailed to below 20%, ischemia and hypoxia induce severe reactions such as ATP exhaustion, invalidation of Na^+^/K^+^ pumps, proliferation of intracellular Ca^2+^, and discharge of neurotoxic substances in the ischemic core region within a short period, which results in speedy cell death. It is surrounded by the peri-infarct region or penumbra, which encloses brain tissue that is functionally damaged but potentially salvageable. Cells in the penumbra region are not electrically active but retain energy in the form of ATP, and thereby the cells are typically enabled to die in a modulated programmed manner, which prevents deleterious inflammation from occurring by rendering cellular contents from being released into the extracellular milieu [55]. Hence, salvaging ischemic penumbral regions and facilitating cell survival are the primary goals of exploiting neuroprotective strategies to mitigate the severity of cerebral ischemic injury through immediate intervention [56].

Recent research has revealed that ferroptosis is an influential factor in brain I/R injury, and its development is closely linked to various biological courses like iron, amino acid, polyunsaturated fatty acid metabolism, and GSH biosynthesis [57]. Morphologically, ferroptosis occurs mainly within the cell, as evidenced by a reduction in mitochondrial volume, density of the bilayer membrane, and reduction or disappearance of the mitochondrial cristae, but with intact membranes and normal nuclear size [58, 59]. Biochemically, ferroptosis is manifested by intracellular GSH depletion, the reduction of GPX4 activity, and the inability of GPX4 to metabolize lipid peroxides to catalyze the reduction reaction, which generates a large amount of ROS and facilitates ferroptosis [60]. Previous studies have indicated that high levels of GPX4 can safeguard neurons and mitochondria from oxidative damage [61, 62]. Meanwhile, reduced levels of GPX4 dramatically diminished the amount of NeuN-positive cells in the hippocampus and elicited neuronal ferroptosis [63, 64], implying that GPX4 is a crucial factor in the modulation of neuronal ferroptosis. Similarly, diminished levels of SLC7A11 can generate a drop in intracellular cystine levels, leading to GSH depletion and inhibition of GPX4 activity, eventually activating ferroptosis [65–67]. Therefore, the expression level of SLC7A11 can directly mediate the susceptibility of cells to ferroptosis. In addition, FTH1 is known to carry out high levels of iron storage, thus ensuring normal biochemical reactions in vivo [68], and the genesis of ferroptosis is often accompanied by reduced levels of FTH1 [69]. Our results showed that the levels of GPX4, SLC7A11, and FTH1 were decreased after MCAO/R, while EA treatment significantly augmented the expression of GPX4, SLC7A11, and FTH1.

Iron is characterized by a diversity of metabolic roles and performs valuable physiological functions in vivo. Extracellular Fe^3+^ combines with transferrin (TF) and is internalized to the nucleus through transferrin receptor 1 (TFR1) on the cell membrane, where Fe^3+^ is reduced to Fe^2+^. Eventually, divalent metal transporter 1 (DMT1) discharges Fe^2+^ from the nuclear endosome into the intracytoplasmic unstable iron pool in the cytoplasm. Furthermore, ferroportin 1 (FPN1) on the cell membrane frees surplus Fe^2+^ to the exterior of the cell to ensure the maintenance of iron homeostasis [70, 71]. While the body's tissues or organs suffer damage, the cells are under stress and the intracellular redox balance is in jeopardy, with a resultant reduction of Fe^3+^ to Fe^2+^. Overloaded Fe^2+^ can catalyze the conversion of H_2_O_2_, a product of oxidative respiration in mitochondria, into hydroxyl radicals, which undergo nonenzymatic lipid peroxidation, eliciting membrane lipid peroxidation and mitochondrial damage, resulting in ferroptosis, which then exacerbates pathological damage [70, 72]. MDA as the terminal product of membrane lipid peroxidation reaction is a biomarker of lipid peroxidation and oxidized protein damage. SOD is among the prominent enzymes capable of scavenging ROS efficiently [73], and is liable for the conversion of superoxide anion, a generated ROS, into hydrogen peroxide, which is then converted to H_2_O by CAT (catalase), thus it is a crucial endogenous antioxidant factor for preserving cellular redox homeostasis [74–76]. Levels of MDA, SOD, and Fe^2+^ have been recognized as signatures and pivotal indicators of ferroptosis; as yet, the definitive mechanisms governing ferroptosis downstream of lipid peroxidation remain elusive. Therefore, while the levels of MDA, SOD, and Fe^2+^ cannot be regarded as the “gold standard” for ferroptosis, for the time being, these indicators can be one of the bases for determining if ferroptosis occurs in cells, and in the future, we will also further seek for more applicable indicators to refine the experiments. We showed that high accumulation of Fe^2+^ in the brain of I/R-injured rats resulted in an imbalance of the antioxidant system, whereas EA treatment remarkably declined MDA levels, elevated SOD activity, and lessened ROS content, pointing to a striking amelioration of intracerebral lipid peroxidation and curtailing of ferroptosis, and that suppressing the origin and progression of cellular ferroptosis might be an equally valuable target for the exploration of novel therapeutic approaches for ischemic stroke in the future.

The brain I/R injury belongs to the category of "stroke disease" in Chinese medicine, its cause is mainly due to the deficiency of healthy energy in the body, which leads to the inability to transport blood, so it stagnates in the body and produces stagnation. Although the vital signs have been stabilized during the recovery period, the blood stasis and phlegm turbidity have not been effectively eradicated [77], thus the treatment is centered on the principle of activating blood circulation, removing blood stasis, and regulating the meridians [78]. Growing evidence indicates that EA is a potential strategy to promote neurological recovery in patients with ischemic stroke. As a safe and effective treatment, EA can be used to alleviate the symptoms of ischemic stroke and promote neurological recovery. Clinical trials and meta-analyses have revealed the effectiveness of EA in alleviating spasticity, reinforcing muscle strength, and achieving better overall health post-stroke, whilst improving patients' quality of life [79]. It has been reported that EA not only protects neurovascular units by modulating cellular autophagy but also significantly reduces the level of oxidative stress and inhibits cellular ferroptosis [80]. Compared to acupuncture alone, EA can produce a synergy effect through combining acupuncture and electrical stimulation [81]. Four acupoints were chosen for this study: Baihui, Dazhui, Neiguan, and Quchi. Baihui and Dazhui have special therapeutic effects on brain disorders, and they are commonly used acupoints for emergencies as recorded in ancient Chinese medical literature, in the preventive period, acute period, relief period, and sequelae period of cerebral stroke, especially in the acute period of cerebral stroke in the state of coma is the preferred acupoints, which are worthy of in-depth study [82]. Some research has found that acupuncture at Baihui and Dazhui points can reduce neuronal apoptosis in the ischemic area, inhibit oxidative stress, attenuate ischemic cerebral edema [83–85], and other mechanisms, that can promote the recovery of neurological function. In addition, acupuncture at the Quchi and Neiguan points has the property of replenishing vital energy and can cure hemiplegia, which are crucial points about health care. When we applied EA stimulation to these four acupoints, it significantly improved neurobehavioral scores and motor-behavioral outcomes after ischemic stroke and reduced cerebral infarct volume. We further confirmed that EA exerts this neuroprotective effect by inhibiting ferroptosis. Interestingly, we discovered that EA facilitated Nrf2 translocation to the nucleus, and thus we pursued further the correspondence between Nrf2 nuclear translocation and the preventive role of EA against stroke.

Nrf2 is ubiquitously present in a broad range of cells and is redox-sensitive [37]. At equilibrium, Nrf2 is bound to Keap1, continuously ubiquitinated by Cul3 E3 ubiquitin ligase, and subtracted by the proteasome, which maintains a high Nrf2 turnover rate. Under stress, Keap1 is oxidized and inactivated, resulting in the stability and translocation of Nrf2 to the nucleus, where it forms a heterodimer with the small Maf proteins, combines with the ARE, and triggers transcription of its target genes [86]. Nrf2 regulates several downstream pathways, including apoptosis, inflammation, oxidative stress, calcium overload load, etc., assisting the body in upholding redox responses. Therefore, stabilizing Nrf2 activity is pivotal to maintaining redox balance and homeostasis in the brain. After a stroke, excess ROS activate Nrf2 [87, 88], and incremental Nrf2 facilitates the expression of antioxidant genes and dampens ROS expression, which ultimately strengthens the mitochondrial antioxidant response [89] and attenuates the destruction of the blood–brain barrier and neurological damage [90]. HO-1, an ıncredibly essential downstream factor of Nrf2, which degrades heme to CO and biliverdin, products that are usually anti-inflammatory and antioxidant [91], and is a major mediator of the salutary effects of Nrf2 [92]. By decomposing free radicals in the body into water and molecular oxygen, the Nrf2/HO-1 signaling pathway serves as a vital mechanism for the body's defense against oxidative stress, mitigating oxidative stress damage and curtailing the production of oxidative products, thereby rendering anti-inflammatory and antioxidant effects [93]. As such, Nrf2 is located at the center of a sophisticated modulatory network that exerts protective effects against cerebral ischemia through multiple mechanisms [94].

Our study found that EA promoted the translocation of Nrf2 from the cytoplasm to the nucleus and resulted in elevated HO-1 levels. To elucidate the protective mechanism of EA on MCAO/R, we applied Brusatol, an inhibitor of Nrf2, to block this pathway. The results showed that Brusatol decreased the content of Total-Nrf2, Nuclear-Nrf2, and HO-1 and decreased the locomotor ability, increased the infarct volume, increased the levels of lipid peroxides and Fe^2+^, inhibited the expression of GPX4, SLC7A11, and FTH1, and reversed the inhibitory effect of EA on ferroptosis. This finding suggests that EA therapy inhibited the occurrence of ferroptosis in cells by facilitating the nuclear translocation of Nrf2 and exerting its antioxidant effects.

In the last decades, research on ischemic encephalopathy models using rodents has yielded very rewarding results, and many neuroprotective measures have been deemed as prospective therapies. Unfortunately, many drugs have mostly failed to show effectiveness in ischemic stroke patients in randomized clinical trials, however. Therefore, translating research findings into clinical practice is quite a challenging task, and we expect EA to become a more efficacious treatment modality in the clinical management of ischemic stroke. Additionally, modulation of iron metabolism and antioxidant pathways to inhibit ferroptosis is an encouraging goal for the future treatment of ischemic stroke. However, more studies are required to elucidate the functional alterations and molecular mechanisms of ferroptosis. In recent years, Nrf2 has become one of the hotspots in the research field due to its potent value in inhibitory inflammatory response and anti-oxidative stress, therefore, the detailed mechanism of EA on the Nrf2/SLC7A11/GPX4 axis needs to be further explored, such as how EA specifically regulates Nrf2 expression and entry into the nucleus to exert a neuroprotective effect. In addition, in vitro experiments are necessary to investigate the effect of activating Nrf2 in ferroptosis, which could help to further elucidate and verify this process. In this experiment, EA inhibited cellular ferroptosis by stimulating Nrf2 nuclear translocation, thus displaying a neuroprotective effect versus acute brain injury, which provides an emerging target for EA to remedy specific mechanisms of ischemic stroke. As our understanding of the mechanisms and pathology of stroke increases, neuroprotective strategies emerge as hopeful treatments. With multi-center, large-sample, well-quality laboratory, and clinical trials, further studies on the molecular mechanisms underlying the neuroprotective effects of electroacupuncture may provide a scientific basis for the selection of acupoints and therapeutic parameters for clinical treatment, as well as a more optimized strategy.

## Conclusion

The present study highlights the efficacy of EA at Baihui, Dazhui, Quchi, and Neiguan on MCAO/R rats, and simultaneously proposes a mechanism involving the promotion of Nrf2 nuclear translocation and activation of the Nrf2/SLC7A11/GPX4 pathway to suppress ferroptosis following ischemic stroke in rat, these suggesting that EA treatment could be applied as an effective therapeutic intervention to improve neurological function. These findings afford prospective therapeutic mechanisms for EA in the treatment of ischemic stroke at Baishui, Dashui, Quchi, and Neiguan, and render valuable doctrinal support for clinical application.

## Data Availability

The data that support the findings of this study are available from the corresponding author upon reasonable request.
